# A Randomized, Double-Blind Study Assessing Changes in Cognitive Function in Indian School Children Receiving a Combination of *Bacopa monnieri* and Micronutrient Supplementation vs. Placebo

**DOI:** 10.3389/fphar.2017.00678

**Published:** 2017-11-17

**Authors:** Tora Mitra-Ganguli, Soumik Kalita, Sakshi Bhushan, Con Stough, James Kean, Nan Wang, Vidhu Sethi, Anuradha Khadilkar

**Affiliations:** ^1^GSK Research and Development Centre, Haryana, India; ^2^Swinburne Centre for Human Psychopharmacology, Swinburne University, Victoria, BC, Australia; ^3^Jehangir Clinical Development Centre Pvt. Ltd., Jehangir Hospital Premises, Maharashtra, India

**Keywords:** *Bacopa monnieri* extract, Brahmi, micronutrient supplementation, cognitive function, working memory, information processing, Indian, school children

## Abstract

Several studies have indicated a chronic cognitive enhancing effect of *Bacopa monnieri* across different ages and cognitive impairment associated with vitamin and mineral deficiencies in children. Therefore, we investigated the effects of 4-month supplementation with a combination of *B. monnieri* extract and multiple micronutrients on cognitive functions in Indian school children aged 7–12 years. This was a randomized, double-blind, parallel design, single-center study in which 300 children were randomized to receive a beverage either fortified with *B. monnieri* and multiple micronutrients (“fortified”) or a non-fortified isocaloric equivalent (“control”) twice-daily for 4 months. Cognitive function was assessed by the Cambridge Neuropsychological Automated Test Battery (CANTAB) administered at baseline, Day 60 and Day 121. The primary endpoint was change in short-term memory (working memory) from baseline in subjects receiving “fortified” vs. “control” beverages after 4 months. Secondary endpoints included sustained attention, episodic memory, and executive function. The “fortified” beverage did not significantly improve short-term memory or any of the secondary outcomes tested relative to the “control” beverage. However, the spatial working memory “strategy” score showed significant improvement on Day 60 (difference between groups in change from baseline: −0.55; *p* < 0.05), but not on Day 121 due to the active intervention. Study products were well-tolerated. Reasons for these unexpected findings are discussed.

## Introduction

Micronutrient deficiencies can impair cognitive performance in children of all ages. Deficiencies adversely affect many aspects of a child's cognitive functions including lower IQ, poor memory, impaired verbal, and non-verbal learning, depression, attention deficit, and delayed processing speed (Sachdev et al., 2005; Eilander et al., 2010; Khor and Misra, 2012). These deficiencies can also lead to poor academic outcomes, lower employment success and even lower life expectancies (Mendez and Adair, 1999; Shariff et al., 2000; Demment et al., 2003; Grantham-McGregor, 2007). School children are at a critical stage of cognitive development as several areas of the brain that are involved in higher-order cognitive function continue to develop and mature until mid-teenage years (Sowell et al., 2004; Lam and Lawlis, 2016). As such, children and adolescents at risk of micronutrient deficiency are particularly vulnerable to impaired cognitive development and function, which can adversely impact their later adult life.

Several studies have suggested that diet may affect a child's neurocognitive development and learning (Mendez and Adair, 1999; Shariff et al., 2000; Grantham-McGregor, 2007). The developing brain requires a range of proteins, vitamins and energy sources to function effectively. These dietary constraints may also affect learning and cognitive processes particularly in environments which may be deficient in any of these substrates (Sachdev et al., 2005; Eilander et al., 2010; Khor and Misra, 2012). Nutritional deficiency is a highly recognized public health issue affecting over 2 billion people worldwide (Initiative, 2004). The most commonly discussed malnutrition issues throughout the world are vitamin A deficiency (VAD), iodine deficiency disorders (IDD), and iron deficiency anemia (IDA) (Ramakrishnan, 2002). Children deficient in Vitamin A are associated with higher mortality rates than those children supplementing vitamin A in their diet (Awasthi et al., 2013). A study showing increases in school attendance following vitamin A supplementation highlights a secondary effect of treatment via not only reducing illness but enabling active learning (Mahawithanage et al., 2007). Iodine deficiency is the leading cause of brain damage as well as a host of other neurological and cognitive disorders (Meletis, 2011; WHO, 2017). Recent randomized clinical trials have demonstrated that iodine supplementation in children and adolescents' leads to improvements in cognitive outcomes in even mildly deficient subjects (Zimmermann et al., 2006; Gordon et al., 2009). A recent meta-analysis regarding oral iron supplementation in iron-deficient children revealed improvements in attention, concentration and IQ (Falkingham et al., 2010). In such cases, iron-dependent dopamine D2 receptors are thought to become impaired in iron-deficient subjects (Pollitt et al., 1985). Studies using animal models have found that dopaminergic, cholinergic, and opiate system interactions may all play a part in cognitive deficiencies (Youdim, 2008). Previous research has demonstrated improvements in cognitive functioning (Kashyap and Gopaldas, 1987), learning (Soemantri, 1989), and memory (Bruner et al., 1996) following iron supplementation in children with IDA (Sanghvi et al., 2007). Despite the positive findings of individual nutrient supplementation, multiple micronutrients have shown to not only reduce the severity and prevalence of common illnesses but also improve the cognitive abilities of developing children to a greater extent than individual nutrients alone (Solon et al., 2003; Vazir et al., 2006; Muthayya et al., 2009; Nga et al., 2011). A recent meta-analysis demonstrated the positive effects of a multi-micronutrient intervention, as opposed to singular micronutrient interventions, on the physical growth of malnourished children (Ramakrishnan et al., 2004). This highlights the importance of considering several nutrients when targeting micronutrient deficient populations. Previous research investigating the effects of food and beverages fortified with micronutrients has reported significant improvements in a number of cognitive domains including non-verbal intelligence (Schoenthaler et al., 2000), mental abilities (Solon et al., 2003), visual recall (Kumar and Rajagopalan, 2007; Manger et al., 2008), measures of intelligence (Nga et al., 2011), short-term working memory (van Stuijvenberg et al., 1999; Kumar and Rajagopalan, 2007; Muthayya et al., 2009), and attention and concentration (Vazir et al., 2006). A recent chronic (12 month) study investigating the effects of a micronutrient intervention alone (iron, zinc, folate, and vitamins A, B-6, B-12, and C) in well-nourished children from Australia and Indonesia found significant improvements in verbal learning and memory (Osendarp et al., 2007). These results were replicated by a research group in India who also found improvements in the short-term memory and fluid reasoning of children aged 6–10 years taking high and low doses of a micronutrient treatment (Muthayya et al., 2009). Research is also investigating the benefits of micronutrient interventions in pre- and post-natal care (Ramakrishnan et al., 2014; Vanhees et al., 2014) on cognition and neurodevelopment in healthy children (Anjos et al., 2013). This research suggests that nutrition influences neurodevelopment and may be modified to improve cognitive development (Bryan et al., 2004). The current treatment intervention contains vitamins A, D, E, B1, B2, B3, B12, B9, C, Biotin, and Pantothenic Acid and minerals iron, iodine, zinc, selenium, copper, magnesium, and calcium which covers a broad spectrum of vitamin and mineral deficiencies.

India has recently experienced a nutrition transition (Griffiths and Bentley, 2001; Misra et al., 2011), resulting in a 7% decrease in carbohydrate-derived and a 6% increase in fat-derived energy. Furthermore, in addition to the historical problem of under-nutrition in India, rising urbanization and economic development have led to both under- and over-weight individuals coexisting in the same population (Griffiths and Bentley, 2001). In addition, because Indian diets are predominantly cereal-based with little variety, the risk of micronutrient deficiency is high, irrespective of bodyweight (Swaminathan et al., 2013). Deficiencies of iron, vitamin A, iodine, B vitamins, and zinc constitute the common types of micronutrient malnutrition affecting millions of children in low-income countries (Khor and Misra, 2012). In 2004, two billion people worldwide were estimated to be at risk of iodine deficiency (Andersson et al., 2007). In 2011, anemia was estimated to affect 42.6% of school-aged children globally with around 50% of cases linked to an iron deficiency (WHO, 2015).

Nutrition can be modified to optimize cognitive development (Bryan et al., 2004), and micronutrient supplementation assists in preventing deficiencies in those at risk of impaired cognitive performance (Huskisson et al., 2007; Lam and Lawlis, 2016). The micronutrients most closely associated with cognitive function are vitamins B1, B2, B3, B6, B12, C, folic acid and minerals such as iron, iodine, and zinc (Huskisson et al., 2007; Lam and Lawlis, 2016). A recent review assessed the role of micronutrient interventions on cognitive performance of children aged 5–15 years in developing countries and reported a beneficial effect of micronutrient interventions for a duration of 3 months to 1 year, using food fortified with multiple micronutrients (MMN). Many of the studies were conducted in rural communities of low socio-economic status, with a high prevalence of undernutrition, including underweight, stunting, and anemia. Although there was a lack of consistency in the impact of micronutrient supplementation on intelligence, long-term mental functions and school examination grades of the children, a beneficial effect of MMN supplementation on short-term memory was consistently reported (Khor and Misra, 2012).

### *Bacopa monnieri* (“Brahmi”)

**Figure 1:**
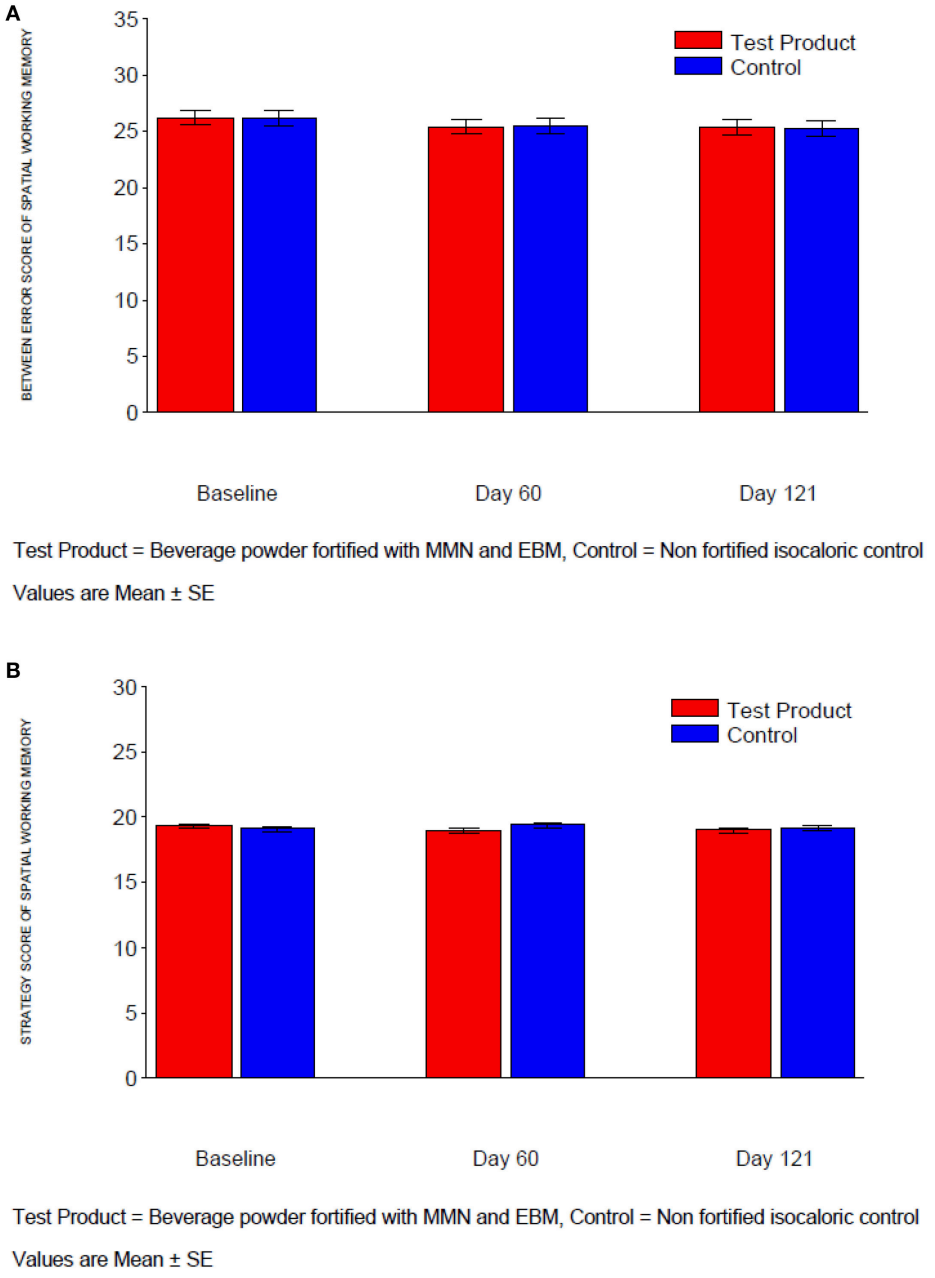
**(A)** Between error score of spatial working memory. **(B)** Strategy score of spatial working memory.

In addition to micronutrients several plant-based extracts have recently been shown to improve cognition. One is the herb *Bacopa monnieri* (L.) Wettst. (syn. *B. monnieri* Hayata & Matsum), or “*Brahmi,”* from the family Scrophulariaceae. Bacopa has been used in the *Ayurvedic* medicinal system for ~3,000 years and is classified as a medhyarasayana, a drug used to improve memory and intellect (medhya) (Russo and Borrelli, 2005). The memory and learning enhancing effects of Bacopa have been studied in healthy adult populations with mostly positive results in terms of cognition (Stough et al., 2001, 2008; Nathan et al., 2004; Pase et al., 2012). A recent systematic review indicated that of nine clinical double blind placebo controlled *B. monnieri* trials in humans, eight demonstrated improvements in memory, attention, cognition, and mood (Pase et al., 2012). These nootropic effects are attributed to the key constituents of Bacopa known as *bacosides A* and *B* (see Figures 1A,B; Das et al., 2002; Pase et al., 2012; Neale et al., 2013). *In-vivo* studies have examined interventions with an extract containing only these bacosides with similar and consistent significant improvements in the areas of memory and learning found (Singh et al., 1988). The mechanisms of action appear to act on the central nervous system and are seen to modulate cholinergic densities (Uabundit et al., 2010), acetylcholine levels (Bhattacharya et al., 1999), have β-amyloid scavenging properties (Holcomb et al., 2006), and demonstrated compelling evidence for its anxiolytic capabilities (Bhattacharya et al., 2000; Russo et al., 2003; Dhanasekaran et al., 2007; Kapoor et al., 2009). The effects of BM on memory, attention, and cognitive function has been less well studies in controlled trials in children. In a non-double-blinded study conducted in 40 children aged 6–8 years from rural India, those receiving Bacopa syrup (350 mg) three times daily for 3 months showed increased exploratory drive, improved perceptual images of patterns and increased perceptual organization and reasoning ability, compared with children who received placebo (Sharma et al., 1987). A randomized, double-blind, placebo-controlled trial evaluating BM in 36 children with diagnosed attention deficit/hyperactivity disorder was conducted over a 16-week period (Negi et al., 2000). In the 19 children who received Bacopa (50 mg twice daily for 12 weeks, followed by 4 weeks' placebo), significantly greater improvements in sentence repetition, logical memory and paired associate learning tasks were observed at 12 weeks compared with the 17 children who received placebo. Furthermore, these improvements were maintained at 16 weeks (after 4 weeks' placebo administration; Negi et al., 2000). A recent review of research into BM in child and adolescent populations reported its potential to improve memory (visual, meaningful, and overall span; Kean et al., 2016). However, despite these encouraging results there remains a paucity of data on the effect of BM on cognitive function in child and adolescent populations. The cognitive benefits of chronic administration of MMN or specific extracts of BM, administered separately, have been reported following 4 months' intervention (Negi et al., 2000; Solon et al., 2003; Nga et al., 2011). This current study investigated the effects of 4 months' supplementation of BM extract (EBM) and MMN (RH01715), administered together, on the cognitive functions of school children aged 7–12 years in India. We hypothesized that a 4-month supplementation with a combination of EBM and MMN would lead to significantly improved cognitive functions, such as memory and attention, compared with a control product.

### Study aims and hypotheses

The primary outcome for this trial was short-term working memory in school children (7–12 years) following 4 months of intervention with RH01715 over controls. Secondary outcomes were executive function, episodic memory, sustained attention, problem solving, intelligence, and reasoning abilities.

## Methods

### RH01715

The treatment intervention RH01715 is a beverage powder containing *multiple micronutrients* (MMN) and a *Bacopa monnieri* extract (EBMl; see Table 1 for a list of ingredients). Based on previous research, the MMN and EBM components of the intervention have been shown to be beneficial for children and adolescents who may be deficient in these nutrients.

**Table 1:** Nutritional profile for study products.

**Nutrients**	MMN/EBM-fortified^*^ per 76 g	Control^*^ per 76 g
**VITAMINS**		
Vitamin A	300 μg	–
Vitamin D	5 μg	–
Vitamin E	5 mg	–
Thiamin (B1)	1.2 mg	–
Riboflavin (B2)	1.3 mg	–
Niacin (B3)	9.6 mg	–
Vitamin B6	1.3 mg	–
Vitamin B12	2.4 μg	–
Folic Acid (B9)	400 μg	–
Vitamin C	40 mg	–
Biotin	12.5 μg	–
Pantothenic Acid	2.5 mg	–
**MINERALS**		
Iron (10% bioavailability)	14.6 mg	–
Iodine	150 μg	–
Zinc	8.6 mg	–
Selenium	16 μg	–
Copper	0.35 mg	–
Magnesium	34.5 mg	–
Calcium	458.5 mg	68.5 mg
**INGREDIENTS**		
Brahmi	130 mg	–
Choline	123.8 mg	–

* *With dairy whitener*.

*EBM, Bacopa monnieri extract; MMN, multiple micronutrients*.

### Study design

This was a randomized, double-blind, parallel design trial comparing the effect of a beverage fortified with MMN and EBM and a non-fortified beverage on short-term memory in children aged 7–12 years old. The study was conducted at a single school in the urban area of Pune, Maharashtra, India, between July 2014 and January 2015. The protocol was approved by an Institutional ethics committee (Jehangir Clinical Development Centre Private Limited; IRB number: ECR/352/Inst/MH/2013). The study was registered at https://clinicaltrials.gov (NCT02416245). Eligible subjects were stratified by age into three strata: aged ≥7 and < 9 years; aged ≥9 and < 11 years; aged ≥11 and < 12 years and were randomized into one of the following two treatment groups: Group 1: Beverage powder fortified with MMN and EBM (“MMN/EBM-fortified”); Group 2: Non-fortified isocaloric beverage powder (“control”).

The MMN/EBM-fortified beverage powder (32 g treatment product plus 6 g dairy whitener; Table 1) and the non-fortified isocaloric beverage powder were both dissolved in 180 mL of lukewarm water and consumed twice daily. The two treatment powders were identical in appearance, presentation, and methods of preparation. The beverages were administered at school, under the supervision of trained research personnel on all school days. In addition, counted number of sachets were dispensed to the subjects to be consumed twice daily under adult supervision at home over the weekend and during other school holidays. Compliance was documented using dispensing logs and counting empty sachets; a subject was considered non-compliant if they consumed ≤ 80% of the total number of sachets over the entire study duration. Each subject consumed the randomly-assigned study beverage twice daily for a total duration of 4 months.

There were fve visits in total: on visits two, three, four, and five, cognitive performance tests were administered in the morning hours when children came to school. Screening was undertaken during Visit 1; Visit 2 was a practice session designed to help subjects become familiarized with the test procedures (up to 14 days post-visit 1); Visit 3 (up to 14 days post-visit 2) denoted Day 1 (baseline); the mid-point assessment was conducted on Day 60 on Visit 4; and Visit 5 occurred on Day 121 and was deemed the end of study. Each subject completed the cognitive performance assessment in ~35–45 min (Table 2), and parallel forms were used to eliminate practice effects.

**Table 2:** Cognitive performance measures.

**Test**	**Test time duration (min)**	**Test description**	**Cognitive function assessed**
Motor control	2	A series of crosses is shown in different locations on the screen. After a demonstration of the correct way to point, using the forefinger of the dominant hand, the participant must touch the crosses in turn.	Familiarization with CANTAB touch screen interface
Spatial Working Memory	7	Subject searches through a series of boxes until they find a blue chip. When they find a blue chip they move it to the “home” area on the right hand side of the screen. They then have to look for the next blue chip which will not be in the same box as before. For each problem, the participant must not return to a box they have previously found a blue chip in. The number of boxes is gradually increased from three to eight boxes. The positions of the boxes used are changed from trial to trial to discourage the use of stereotyped search strategies.	Working memory and executive function
Paired Associate Learning	8	Boxes are displayed on the screen and open up in a randomized order to reveal a number of patterns. The participant must remember which pattern is in which box. The number of patterns gradually increases up to a number of eight but the participant has multiple attempts to remember each location.	Episodic memory
Stockings of Cambridge	11	The subject is shown two displays containing three colored balls. The displays are presented so they are easily perceived as stacks of colored balls held in stockings or socks suspended from a beam. The subject must use the balls in the lower display to copy the pattern shown in the upper display. The balls may be moved one at a time by touching the required ball, then touching the position to which it should be moved. The time taken to complete the pattern and the number of moves required are taken as measures of the subject's planning ability.	Executive functioning
Rapid Visual Information processing	7	The screen displays a box which randomly flashes numbers between 2 and 9. The participant must press the button on the screen to respond when they see a target sequence of 3-5-7.	Sustained attention

*CANTAB, Computerized Neuropsychological Test Automated Battery*.

### Study population

The study population comprised healthy children aged between 7 and 12 years. Children were included if they were willing to comply with all study procedures and able to comprehend at least one of the languages used for cognitive assessment (English, Hindi, or Marathi), had no clinically significant and relevant abnormalities in medical history or upon physical examination and Z-scores of body mass index (BMI) for age of > −2 to < +1. Subjects were only included if they and their parent/legal guardian provided written informed consent at screening after adequate explanation of the aims, methods, objectives, and potential hazards of the study.

Exclusion criteria included being in care; intolerance or hypersensitivity to study materials, or any known food allergies; severe anemia with hemoglobin < 8 g/dL (WHO, 2015); diagnosis of attention deficit hyperactivity disorder (ADHD), reading dyslexia or any other behavioral disorder; participation in another clinical study or receipt of an investigational drug within 30 days prior to the screening visit, or within any nutritional study within 6 months prior to the screening visit; current or relevant history of any serious, severe or unstable physical or psychiatric illness or any medical disorder that would make the subject unlikely to fully complete the study; health conditions that would affect food metabolism, such as food allergies, kidney disease, liver disease, and/or gastrointestinal diseases.

### Statistical analysis

In terms of improvement in working memory, with a sample size of 220 subjects (*n* = 110 per group), the study had 83% power to detect a mean difference of 0.6 (times of revisit) between two treatments using a two-tailed 5% level *t*-test, assuming a standard deviation of 1.5 (on change from baseline; Wesnes et al., 2000, 2012). The sample size was determined as 300 randomized subjects to ensure 220 subjects completed the study. Efficacy on working memory was measured by scores on the “Spatial Working Memory” task of the Computerized Neuropsychological Test Automated Battery (CANTAB) battery' (Table 2). “Sustained attention” was measured by CANTAB task “Rapid Visual Information Processing.” “Episodic memory” was measured by CANTAB task “Paired Associates Learning.” “Executive function” was measured by CANTAB task “Stockings of Cambridge”. “Reasoning ability” was measured by Raven's Colored Progressive Matrices, a non-verbal visual test, that assesses the ability for non-verbal and abstract reasoning (Raven, 2007). All tests are described in Table 2.

The primary variable was spatial working memory “Between Errors” change from baseline in working memory assessment. Secondary variables included changes from baselines in Spatial Working Memory “Strategy;” Rapid Visual Information Processing “Prime,” “Latency,” and “Total False Alarms;” Paired Associates Learning “Total Error (adjusted);” Stockings of Cambridge “Number of Problems Solved;” Raven's Colored Progressive Matrices “Number of Correct Answers.” ANCOVA (Analysis of Covariance) was applied to each of these variables at each post- baseline visit with treatment, age stratum and treatment^*^age interaction as factors and baseline as covariate. For the primary efficacy variable, treatment comparison after 4 months of treatment was of key interest. Estimation on treatment difference and 95% confidence interval (CI) were provided. Treatment comparison per age strata are only provided if the *p* value for treatment^*^age interaction (*F*-test) was ≤ 0.10. Treatment comparisons for all secondary variables were performed. However, there was no multiplicity adjustment applied to these comparisons due to the exploratory nature of the study.

Efficacy assessments were conducted in the intent-to-treat (ITT) population which comprised subjects who were randomized, received study treatment and had at least one post-baseline efficacy measurement. The per protocol (PP) population included all ITT subjects who had at least one assessment of efficacy considered unaffected by protocol violations. Safety assessments were conducted in the safety population which comprised subjects who were randomized and received at least one dose of study treatment.

## Results

### Demographic and baseline characteristics

A total of 310 subjects were screened, and 300 subjects were randomized. Of the 10 subjects not randomized, 9 did not meet the study eligibility criteria and one was lost to follow-up. The safety population comprised 300 subjects (MMN/EBM-fortified: *n* = 149; control: *n* = 151) and 290 subjects were included in the ITT population (MMN/EBM-fortified: *n* = 146; control: *n* = 144). A total of 283 subjects completed the study and were included in the PP population. Of the 290 subjects in the ITT population, all were Asian, 54% were male and age ranged from 7 to 12 years with a mean of 9.3 years. Gender, race and age strata distributions for the safety population and the PP population were similar to that of the ITT population (Table 3).

**Table 3:** Patient demographics and baseline characteristics (ITT population).

	**MMN/EBM-fortified (*n* = 146)**	**Control (*n* = 144)**
**GENDER**, ***N*** **(%)**		
Male	83 (56.8)	74 (51.4)
Female	63 (43.2)	70 (48.6)
**RACE**, ***N*** **(%)**		
Asian	146 (100)	144 (100)
Black or African	0 (0)	0 (0)
American	0 (0)	0 (0)
White		
**AGE, YEARS**		
Mean (*SD*)	9.3 (1.4)	9.3 (1.5)
Median	10.0	9.0
Range	7–12	7–12
**AGE STRATA**, ***N*** **(%)**		
≥7 and < 9	43 (29.5)	44 (30.6)
≥9 and < 11	73 (50.0)	71 (49.3)
≥11 and ≤ 12	30 (20.5)	29 (20.1)

*EBM, Bacopa monnieri extract; ITT, intent-to-treat; MMN, multiple micronutrients; SD, standard deviation*.

### Efficacy results

The primary population for efficacy analysis was the ITT population.

#### Spatial working memory

For measures “Between Error” and “Strategy” in Spatial Working Memory, there was no significant treatment difference after 4 months (Table 4, Figures 1A,B). Similarly, there was no significant treatment difference at Day 60 for “Between Error.” Although the treatment difference at Day 60 for “Strategy” was statistically significant and favored the MMN/EBM-fortified beverage (difference: −0.55; *p* < 0.05), this was not considered clinically significant given the size of the effect.

**Table 4:** Adjusted means^*^ of change from baseline and treatment comparisons of all variables (ITT Population).

**Variable**	**Visit**	**MMN/EBM-fortified (*****N*** = **146)**		**Control (*****N*** = **144)**		**Comparison: MMN/EBM-fortified vs. control beverage**		
		***N***	**Mean (SE)**	***N***	**Mean (SE)**	**Difference**	**95% CI**	***P* value**
SWM	Day 60	146	−0.63 (0.55)	144	−0.65 (0.55)	0.02	(−1.51, 1.56)	0.976
“Between Error”	Day 121	142	−0.78 (0.60)	141	−0.87 (0.60)	0.1	(−1.57, 1.77)	0.907
SWM	Day 60	146	−0.26 (0.18)	144	0.29 (0.18)	−0.55	(−1.04, −0.06)	0.029
“Strategy”	Day 121	142	−0.26 (0.19)	141	0.04 (0.19)	−0.30	(−0.82, 0.22)	0.250
RVIP	Day 60	145	−0.00 (0.00)	144	−0.00 (0.00)	−0.00	(−0.01, 0.01)	0.887
“Prime”	Day 121	141	−0.01 (0.00)	141	0.00 (0.00)	−0.01	(−0.02, 0.00)	0.141
RVIP	Day 60	145	−14.71 (7.14)	144	−26.09 (7.14)	11.38	(−8.49, 31.25)	0.261
“Median latency”	Day 121	141	−21.79 (8.02)	141	−35.35 (8.00)	13.57	(−8.72, 35.86)	0.232
RVIP	Day 60	145	1.43 (1.05)	144	1.91 (1.05)	−0.48	(−3.41, 2.45)	0.748
“False alarm”	Day 121	141	2.28 (1.37)	141	2.74 (1.36)	−0.45	(−4.25, 3.34)	0.814
Paired Associates Learning	Day 60	146	0.59 (0.86)	144	1.38 (0.87)	−0.79	(−3.19, 1.61)	0.516
	Day 121	142	0.62 (0.78)	141	0.93 (0.78)	−0.32	(−2.49, 1.86)	0.773
Stockings of Cambridge	Day 60	145	0.33 (0.15)	140	0.35 (0.15)	−0.02	(−0.43, 0.39)	0.908
	Day 121	141	0.23 (0.15)	136	0.34 (0.15)	−0.11	(0.52, 0.31)	0.613
RCPM	Day 60	146	1.29 (0.39)	144	1.41 (0.39)	−0.13	(−1.21, 0.96)	0.819
	Day 121	142	1.60 (0.43)	141	1.84 (0.43)	−0.24	(−1.45, 0.97)	0.700

* *From ANCOVA model with treatment, age strata, and treatment age interaction as factors and baseline as covariate*.

*ANCOVA, Analysis of Covariance; CI, confidence interval; EBM, Bacopa monnieri extract; ITT, intent-to-treat; MMN, multiple micronutrients; RCPM, Raven's Coloured Progressive Matrices; RVIP, Rapid Visual Information Processing; SE, standard error; SWM, Spatial Working Memory*.

#### Rapid visual information processing

**Figure 2:**
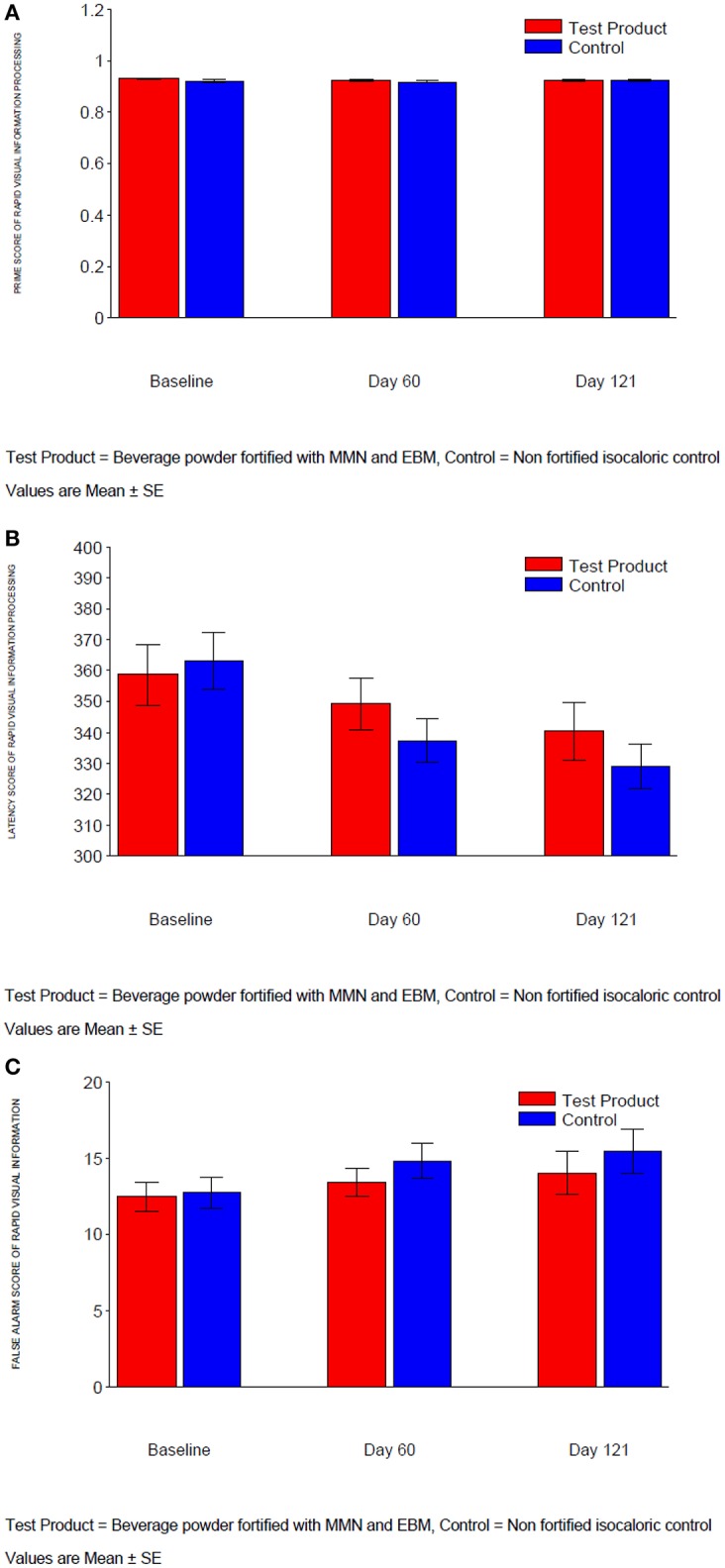
**(A)** Prime score of rapid visual information processing. **(B)** Latency of rapid visual information processing. **(C)** False alarm of rapid visual information processing.

For measures “Prime,” “Median latency,” and “Total false alarm” in Rapid Visual Information Processing, there were no significant treatment differences at either Day 60 or Day 121 (Table 4, Figures 2A–C). At Day 121, *p*-values for treatment by age strata interaction in the ANCOVA for “Prime” and “Total false alarm” were < 0.1 (*p* = 0.031 and *p* = 0.076, respectively); as such, treatment comparisons at Day 121 for these two endpoints were made for each age stratum (Table 5).

**Table 5:** Adjusted means^*^ of change from baseline and treatment comparisons for Rapid Visual Information Processing (“Prime” and “False Alarm”) scores according to age stratum at Day 21 (ITT Population).

**Variable**	**Age stratum**	**MMN/EBM-fortified**		**Control**		**Comparison: MMN/EBM-fortified vs. control beverage**		
		***N***	**Mean (SE)**	***N***	**Mean (SE)**	**Difference**	**95% CI**	***P* value**
RVIP “Prime”	≥7 and < 9 years	42	−0.01 (0.01)	44	0.01 (0.01)	−0.02	(−0.04, −0.00)	0.024
	≥9 and < 11 years	70	0.00 (0.01)	68	−0.01 (0.01)	0.01	(−0.01, 0.02)	0.217
	≥11 and ≤ 12 years	31	−0.01 (0.01)	29	0.00 (0.00)	−0.01	(−0.02, 0.00)	0.141
RVIP “False alarm”	≥7 and < 9 years	42	0.94 (2.37)	44	6.84 (2.30)	−5.91	(−12.37, 0.56)	0.073
	≥9 and < 11 years	70	0.07 (1.82)	68	1.41 (1.85)	−1.34	(−6.45, 3.77)	0.606
	≥11 and ≤ 12 years	29	5.84 (2.84)	29	−0.04 (2.85)	5.89	(−1.98, 13.75)	0.142

* *From ANCOVA model with treatment, age strata, and treatment age interaction as factors and baseline as covariate*.

*CI, confidence interval; EBM, Bacopa monnieri extract; ITT, intent-to-treat; MMN, multiple micronutrients; RVIP, Rapid Visual Information Processing; SE, standard error*.

For “Prime,” the treatment response had the similar pattern in stratum 1 (age ≥7 and < 9 years) and stratum 3 (age ≥11 and ≤ 12 years) with around 0.01 decrease for the MMN/EBM-fortified group and 0.01 increase after treatment in control group. However, the reverse was seen for stratum 2 (age ≥9 and < 11 years) with no change in MMN/EBM-fortified group and 0.01 decrease in control group, suggesting significant interaction (*p* < 0.1). For “Total false alarm,” younger children in the control group tended to make ~6 more errors after 4 months of treatment than those in the MMN/EBM-fortified group; in contrast, older children in the MMN/EBM-fortified group tended to make ~6 more errors after 4 months of treatment than those in control group. This contrasting pattern in the two strata caused significant interaction to be detected for “Total false alarm.” However, the interaction was not detected at Day 60, and not detected for other CANTAB measures. In addition, the magnitudes of treatment responses (0.01 in “Prime,” 6 in “Total false alarm”) were very small and therefore not considered to be clinically relevant.

#### Paired associates learning, stockings of cambridge and raven's colored progressive matrices

**Figure 3:**
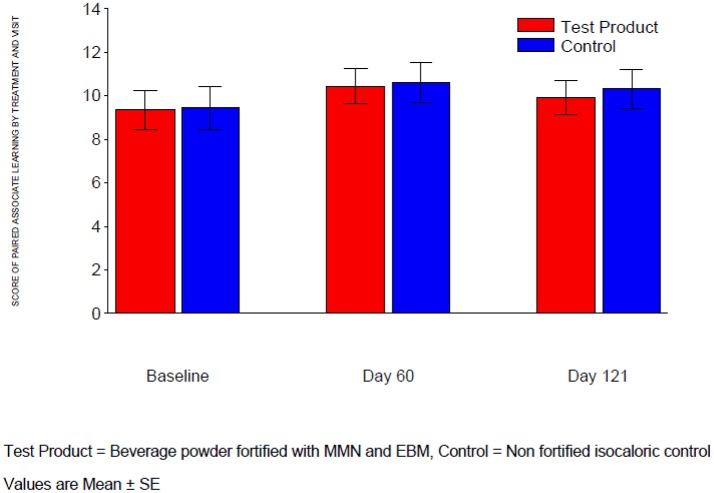
Total error (adjusted) of paired associates learning.

**Figure 4:**
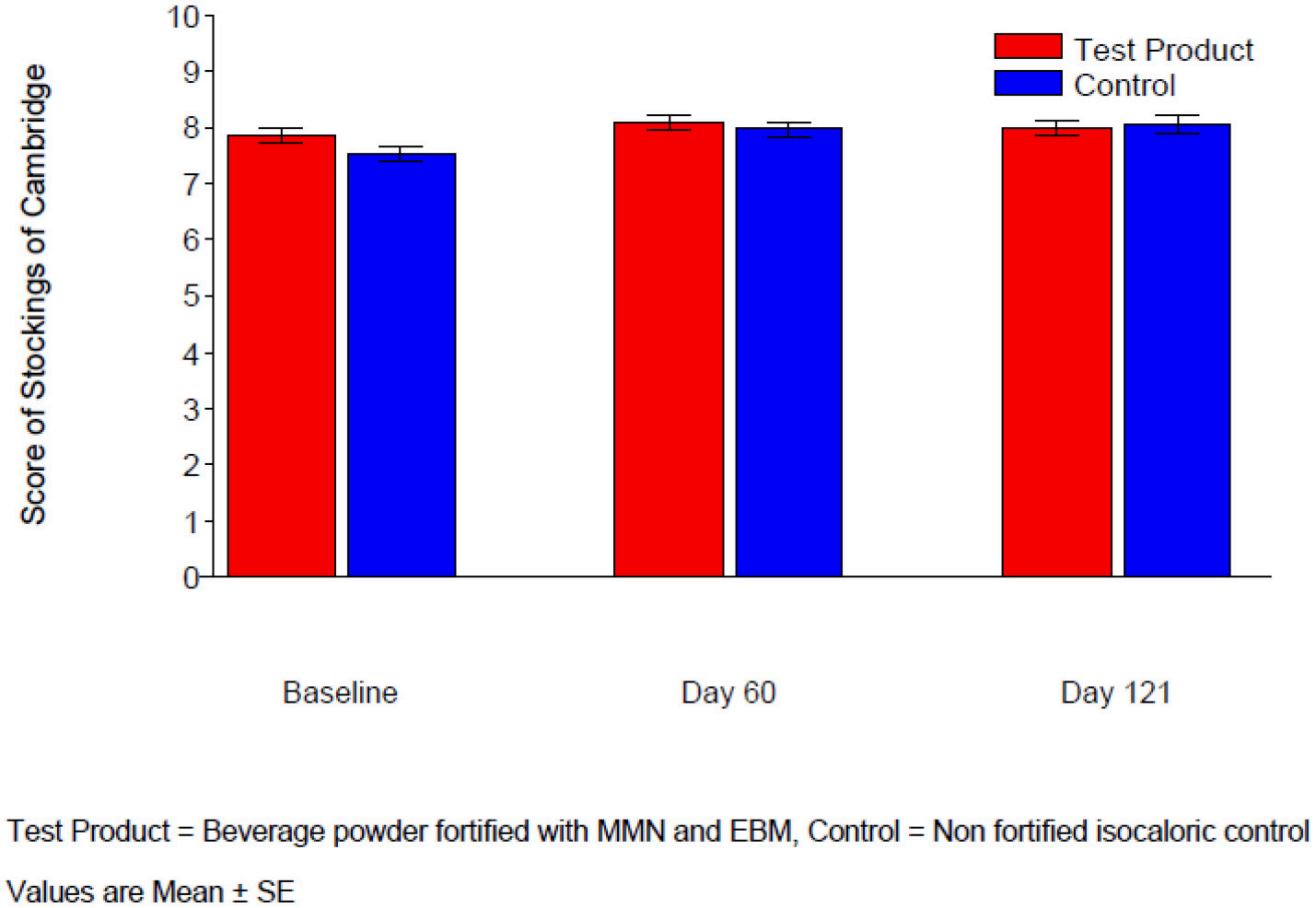
Score of stockings of Cambridge.

**Figure 5:**
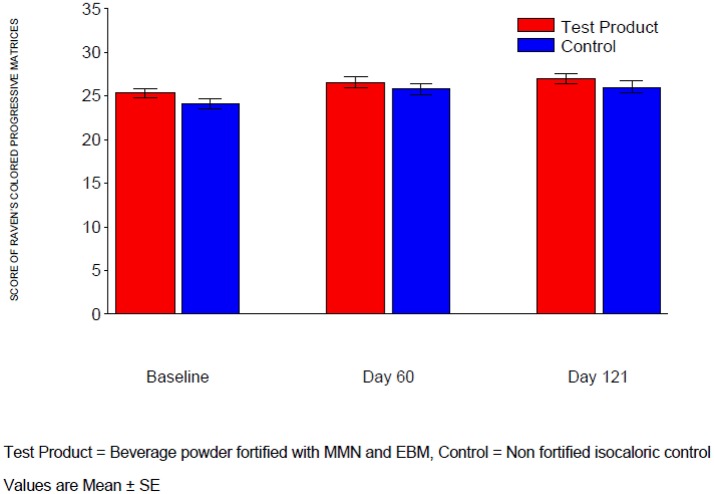
Score of Raven's coloured progressive matrices.

For the measures “Total Error (adjusted)” in Paired Associates Learning, “Problems Solved in Minimum Moves” in Stockings of Cambridge and “number of correct answers” in Raven's colored progressive matrices, there was no significant treatment difference at either Day 60 or Day 121 (Table 4, Figures 3–5). The *p*-value for treatment by age strata interaction in the ANCOVA analysis of either visit was >0.1, therefore treatment comparisons by age strata were not made.

### Safety results

In total 17 subjects in the MMN/EBM-fortified group reported 22 treatment emergent AEs and 24 subjects in the control product group reported 30 treatment emergent AEs (Table 6). There were no treatment-emergent treatment related AEs, and no serious adverse events were reported.

**Table 6:** Treatment emergent adverse events (safety population).

	**MMN/EBM-fortified (*****N*** = **149)**		**Control (*****N*** = **151)**	
	***n* (%)**	**nAE**	***n* (%)**	**nAE**
Number of subjects with at least one AE	17 (11.4)	22	24 (15.9)	30
Gastrointestinal disorders	9 (6.0)	13	13 (8.6)	18
Infections and infestations	4 (2.7)	4	6 (4.0)	6
General disorders/Administration site	2 (1.3)	2	3 (2.0)	3
Respiratory, thoracic and mediastinal disorders	3 (2.0)	3	2 (1.3)	2
Nervous system disorders	0 (0)	0	1 (0.7)	1

*AE, adverse event; EBM, Bacopa monnieri extract; MMN, multiple micronutrients n, number of subjects; nAE, number of adverse events*.

## Discussion

The impact of micronutrients on cognitive functions in school aged children remains unclear. It has been proposed that multiple micronutrients may have a synergistically positive influence on the hippocampus, an area known to play a key role in the encoding and retrieval of novel information in short term memory (Kumaran, 2008). Although a review of micronutrient intervention studies reported a beneficial effect of using food fortified with multiple micronutrients on short term memory, it was suggested that additional RCTs be conducted in developing countries to provide more definitive evidence of the impact of micronutrients on improving cognition in school-aged children (Khor and Misra, 2012) particularly in environments in which deficiencies occur. The botanical product, BM, has been used in traditional medicine as a “memory tonic” (Russo and Borrelli, 2005) although its mechanism of action in children is unknown. The cognitive benefits of chronic administration of MMN or extracts of BM, administered separately, have been reported following 4 months' intervention in three studies in children (Negi et al., 2000; Solon et al., 2003; Nga et al., 2011) and in numerous studies in adults.

The current study compared the effects of a 4-month supplementation with a combination of MMN and EBM fortified beverage with a non-fortified beverage on the cognitive functions of healthy school children aged 7–12 years in India. In our study, we showed that the beverage powder fortified with MMN and EBM extract did not significantly affect either short term memory or any of the secondary outcomes tested. Although the SWM strategy score showed significant improvement on Day 60, there was no statistical significance when tested on Day 121. With regard to rapid visual information processing, although there were no significant treatment differences observed on either Day 60 or Day 121, disparities in “Prime” and “Total false alarm” were seen across the age stratums. Improved “prime” function with the EBM and MMN fortified beverage was observed in the youngest (age ≥7 and < 9 years) and oldest (age ≥11 and ≤ 12 years) stratums, while the reverse was seen in stratum 2 (age ≥9 and < 11 years), suggesting an interesting interaction. For “Total false alarm,” after 4 months of treatment, younger children in the control group made more errors than those who received the MMN/EBM-fortified beverage; in contrast, in older children, error rates were higher in the active treatment vs. the control group. This divergent pattern suggests a significant interaction for “Total false alarm;” however, the magnitudes of treatment responses were not considered to be clinically relevant. Therefore, it is likely that the two interactions detected were due to chance (type 1 error) rather than indicative of a true underlying treatment-by-age interaction.

While micronutrient interventions have been found to improve children's mental development in participants deficient in certain micronutrients, such as iron (Sachdev et al., 2005) and iodine (Bleichrodt and Born, 1994), in our study, the failure of the combined MMN and EBM supplement to significantly improve cognitive function is consistent with some but not all previous reports in healthy children. A meta-analysis of MMN supplementation in healthy children aged 5–16 years failed to show a significant effect on short term memory (Eilander et al., 2010). In their recent meta-analysis of RCTs, Lam and colleagues reported inconsistent findings regarding the impact of micronutrient supplementation and the cognitive domains of short-term memory and attention and that micronutrient supplementation had no significant effect on school performance (Lam and Lawlis, 2016). While the specific interrelationship of diet, brain development and cognition is complex, micronutrients are known to have a direct influence on cognitive function through their involvement in the energy metabolism of neurons and glia cells, neurotransmitter synthesis, receptor binding etc (Simeon and Grantham-McGregor, 1990; Huskisson et al., 2007). Current preclinical research has demonstrated various central nervous system actions of BM including antioxidant (Bhattacharya et al., 2000), anti-depressant (Sairam et al., 2002), and nootropic (Hota et al., 2009) effects; along with direct and indirect links to changes in dopamine (DA), serotonin (5-HT), noradrenaline (NA) and acetylcholine (ACh) neurotransmitter systems (Charles et al., 2011). It is possible that the dosage of EBM used in this study might have been too low to effects these central nervous system actions that could ultimately have a positive impact on attention and memory.

In our study, the choice of tests was guided by two principles: (i) tests that were validated in Indian children and (ii) were translated into Indian languages. The CANTAB system was employed to evaluate cognitive performance. It is a computerized test battery that consists of a series of neuropsychological tests specific to particular aspects of sustained attention, working memory, executive function (planning), and episodic memory. This battery was developed to identify both the neural and neurochemical substrates of cognitive parameters in children. The CANTAB tests have been widely used in interventional drug trials and nutrition studies and have been validated in various populations: children, young adults and the elderly (Sheppard and Cheatham, 2013). The CANTAB tests are available in Hindi, and Marathi. The Raven's Progressive Colored Matrices (Raven, 2007) was used to test for other executive functions (problem solving and reasoning ability) and is a well-validated measure of basic cognitive functioning (Raven, 2000).

The beverage powder fortified with MMN and BME was well-tolerated by the children, with no serious adverse events observed. Our observations are in agreement with studies in which high dosages of BM have been administered in children with no major side effects (Sharma et al., 1987; Dave et al., 2008).

Any conclusions drawn from our findings must be made within the context of the study's limitations and this is particularly true for non-significant treatment effects. A range of potential methodological issues may have contributed to the lack of an effect due to the treatment: (1) The dose of BM extract used in our study (260 mg/day) was lower than that used in previous studies that have reported significant cognitive effects (Sharma et al., 1987; Ramarao et al., 2011). Future studies could include higher doses of Bacopa, such as those used by Sharma et al. (1,050 mg Bacopa per day; Sharma et al., 1987) and Ramarao et al. (2,000 mg of Bacopa per day; Ramarao et al., 2011); (2) There is also a possibility that the bioavailability of Bacopa may have been reduced in a food supplement matrix or in a beverage powder rather than a capsule. Previous studies have used Bacaopa extract in the form of a capsule (Negi et al., 2000) or syrup (Ramarao et al., 2011), and it would be of interest to compare whether the efficacy of Bacopa differs according to method of administration as this may affect bioavailability; (3) Another shortcoming related to the potential confounding effect of the overall diet of the children which may have significantly differed between participants. In addition, a general lack of energy and protein in the diets of children in developing countries such as India, could have overridden the effect of micronutrients; (4) It is also possible that the duration of the trial may have been too short to demonstrate beneficial effects. longer term studies are needed to further elucidate the role of micronutrient supplementation on children's cognitive function. In addition, in a healthy child population, the impact of supplementation with MMN and EBM is likely to be less discernible and without a food frequency questionnaire at baseline or blood samples that are able to determine micronutrient deficiencies it is not possible to know whether the children were deficient or otherwise healthy in key nutrients supplemented in this study; (5) lastly it is unknown how MMN and EBM interacts and whether the simultaneous administration of these substances interact in some unknown manner. Future studies may wish to address some of these shortcomings and evaluate whether there are sub-groups related to such variables as diet, SES, scholastic performance, diet, and BMI.

In summary, this study showed that when compared with a non-fortified formulation, a beverage powder fortified with multiple micronutrients and *B. monnieri* extract did not have a significant impact on either short term memory or any of the secondary outcomes tested in the Indian school children tested.

## Author contributions

TM-G, CS, JK, SB, and SK were responsible for study design and inputs in protocol writing. TM-G wrote the paper, while NW performed the statistical analyses. VS was responsible for the overall conduct of the study. As the Principal Investigator, AK was responsible for the conduct of the study.

### Conflict of interest statement

This study was conceptualized and funded by GSK with the scientific input of Swinburne University in Melbourne. Financial aid was given to the Jehangir Clinical Development Centre to independently collect the data. Data analysis was conducted by both GSK and Swinburne University independently.
